# Performance of ChatGPT-3.5 and ChatGPT-4 in the Taiwan National Pharmacist Licensing Examination: Comparative Evaluation Study

**DOI:** 10.2196/56850

**Published:** 2025-01-17

**Authors:** Ying-Mei Wang, Hung-Wei Shen, Tzeng-Ji Chen, Shu-Chiung Chiang, Ting-Guan Lin

**Affiliations:** 1 Department of Medical Education and Research, Taipei Veterans General Hospital Hsinchu Branch, 81, Section 1, Zhongfeng Road, Zhudong, Hsinchu, 310, Taiwan, 886 03-5962134 ext 127; 2 Department of Pharmacy, Taipei Veterans General Hospital Hsinchu Branch, Hsinchu, Taiwan; 3 School of Medicine, National Tsing Hua University, Hsinchu, Taiwan; 4 Hsinchu County Pharmacists Association, Hsinchu, Taiwan; 5 Department of Family Medicine, Taipei Veterans General Hospital Hsinchu Branch, Hsinchu, Taiwan; 6 Department of Family Medicine, Taipei Veterans General Hospital, Taipei, Taiwan; 7 Department of Post-Baccalaureate Medicine, National Chung Hsing University, Taichung, Taiwan; 8 Institute of Hospital and Health Care Administration, School of Medicine, National Yang Ming Chiao Tung University, Taipei, Taiwan

**Keywords:** artificial intelligence, ChatGPT, chat generative pre-trained transformer, GPT-4, medical education, educational measurement, pharmacy licensure, Taiwan, Taiwan national pharmacist licensing examination, learning model, AI, Chatbot, pharmacist, evaluation and comparison study, pharmacy, statistical analyses, medical databases, medical decision-making, generative AI, machine learning

## Abstract

**Background:**

OpenAI released versions ChatGPT-3.5 and GPT-4 between 2022 and 2023. GPT-3.5 has demonstrated proficiency in various examinations, particularly the United States Medical Licensing Examination. However, GPT-4 has more advanced capabilities.

**Objective:**

This study aims to examine the efficacy of GPT-3.5 and GPT-4 within the Taiwan National Pharmacist Licensing Examination and to ascertain their utility and potential application in clinical pharmacy and education.

**Methods:**

The pharmacist examination in Taiwan consists of 2 stages: basic subjects and clinical subjects. In this study, exam questions were manually fed into the GPT-3.5 and GPT-4 models, and their responses were recorded; graphic-based questions were excluded. This study encompassed three steps: (1) determining the answering accuracy of GPT-3.5 and GPT-4, (2) categorizing question types and observing differences in model performance across these categories, and (3) comparing model performance on calculation and situational questions. Microsoft Excel and R software were used for statistical analyses.

**Results:**

GPT-4 achieved an accuracy rate of 72.9%, overshadowing GPT-3.5, which achieved 59.1% (*P*<.001). In the basic subjects category, GPT-4 significantly outperformed GPT-3.5 (73.4% vs 53.2%; *P*<.001). However, in clinical subjects, only minor differences in accuracy were observed. Specifically, GPT-4 outperformed GPT-3.5 in the calculation and situational questions.

**Conclusions:**

This study demonstrates that GPT-4 outperforms GPT-3.5 in the Taiwan National Pharmacist Licensing Examination, particularly in basic subjects. While GPT-4 shows potential for use in clinical practice and pharmacy education, its limitations warrant caution. Future research should focus on refining prompts, improving model stability, integrating medical databases, and designing questions that better assess student competence and minimize guessing.

## Introduction

### Background

With the advent of the artificial intelligence (AI) era, applications of AI in the medical field have increased with ChatGPT (OpenAI) being the most notable examples. ChatGPT is a large language model based on a generative pretrained transformer developed by OpenAI. ChatGPT-3.5 (GPT-3.5) was the first publicly accessible version, while ChatGPT-4 (GPT-4) was the subscription version. GPT-4 surpasses GPT-3.5 in advanced reasoning, almost nearing human-level performance in professional and academic examinations [1,2]. For instance, GPT-4 ranked in the top 10% of scores on a law examination, whereas GPT-3.5 ranked in the bottom 10% [3]. Additionally, GPT-3.5 resolved 90% of false-belief tasks, achieving the level of a 7-year-old child, whereas GPT-4 resolved 95% of these tasks [4]. Following its launch, ChatGPT has been extensively studied and discussed in both the medical and educational fields [5]. The most widely recognized performance of GPT-3.5 has been on the United States Medical Licensing Examination (USMLE) [6,7]; however, GPT-3.5’s performance did not meet expectations in other examinations [8-11]. Gradually, Nori et al [12]observed that the accuracy of GPT-4 was higher than that of the GPT-3.5 on the USMLE, and further studies confirmed that GPT-4 outperforms GPT-3.5 [13-16]. However, there has been limited research on its performance in pharmacy examinations.

In the field of pharmacy, GPT-3.5 has exhibited commendable performance in clinical toxicology and pharmacology [17,18], although it has not passed the National Pharmacist Licensing Examination (NPLE) in Taiwan [19]. However, GPT-4 has outperformed GPT-3.5 in drug information [20] and China’s Pharmacist Licensing Examination [21]. Generative AI models, a large language model, has been applied in drug development and novel drug design [22-24], pharmacovigilance [25,26], pharmacokinetic model development [27], pharmacy education, and research writing [28,29].

### Goal of the Study

According to previous studies, GPT-3.5 failed to pass the NPLE, indicating its limitations in pharmacy education. Based on these findings, we hypothesized that GPT-4 would outperform GPT-3.5 in this context, demonstrating greater proficiency. To test this hypothesis, this study compared the performance of GPT-3.5 and GPT-4 on Taiwan’s NPLE. Additionally, we conducted a comprehensive assessment of their performance across various question types, with a focus on pharmacy-related tasks such as pharmacokinetic calculation and clinical decision-making scenarios. This analysis aims to determine the practical applications of GPT-4 in pharmacy education and establish guidelines for its optimal use in this field.

## Methods

### Background

The NPLE in Taiwan is divided into 2 stages. The first stage focuses on 3 basic subjects: pharmacology and pharmaceutical chemistry, pharmaceutical analysis and pharmacognosy (including traditional Chinese medicine), and pharmaceutics and biopharmaceutics. The second stage focuses on 3 clinical subjects: dispensing and clinical pharmacy, pharmacotherapy, and pharmacy administration and pharmacy law. The first and second stages of the examination have 240 and 210 multiple-choice questions, respectively. Pharmacy students typically complete the first-stage exam after completing their third year of university coursework. They become eligible for the second-stage exam only after passing the first examination, completing their internships and obtaining their graduation certificates. After passing the second-stage examination, candidates receive their pharmacist certificate, allowing them to practice as a pharmacist legally.

### Data Source

This study used the 2-stage NPLE questions released by the Ministry of Examination in February 2023, with each subject exam lasting for 1 hour. The version of NPLE used in this study was the most recent available at the time of research. We used both GPT-3.5 (free version) and GPT-4 (licensed version). No temperature settings were applied. Examination questions were manually fed into GPT-4 and GPT-3.5 sequentially. To simulate student responses, complete questions were entered into the models without tailored prompts. One question was input at a time, and the responses were recorded for analysis. Since GPT-3.5 cannot process images and image functionality of GPT-4 was unavailable during the analysis, only text-based questions were used. Questions containing graphics, such as chemical structures, tables, symbols, and formulas were excluded. Both models were presented with the same set of questions under identical conditions. Due to the limitations on the number of times the model could be used and required cooling time between queries, all questions were answered sequentially and not timed to avoid any potential bias introduced by time constraints.

### Study Design

**Figure 1.**
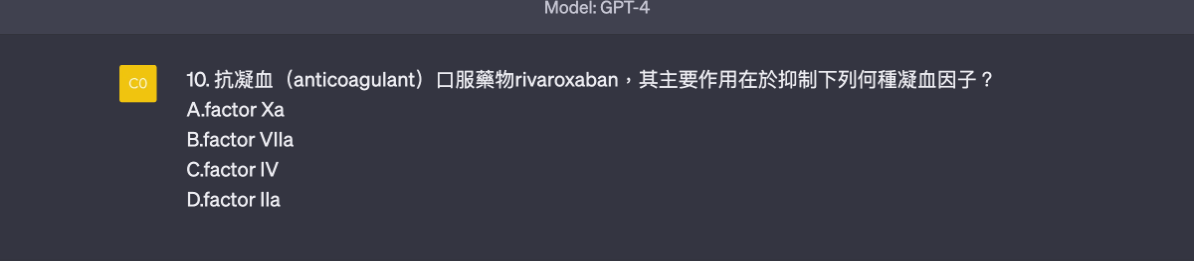
Template of a memory-based question (choose 1 correct word from 4 options, requiring low-level thinking).

**Figure 2.**
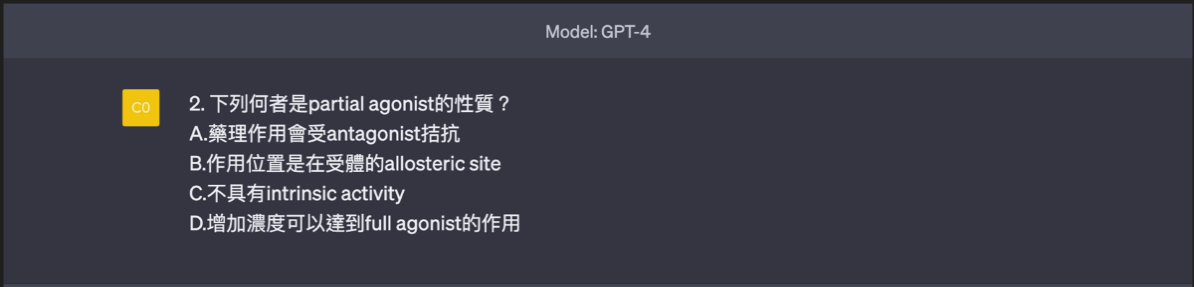
Template of a judgment question (choose 1 correct statement from 4 options, requiring medium-level thinking).

**Figure 3.**
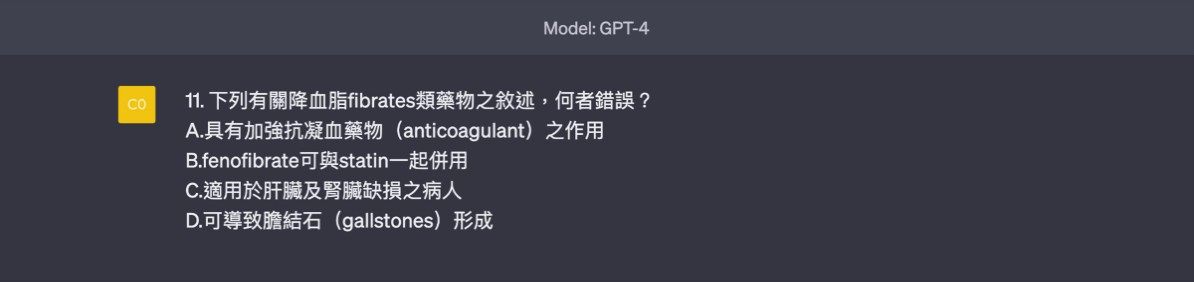
Template of a reverse question (choose 1 incorrect statement from 4 options, requiring medium- to high- level thinking).

**Figure 4.**
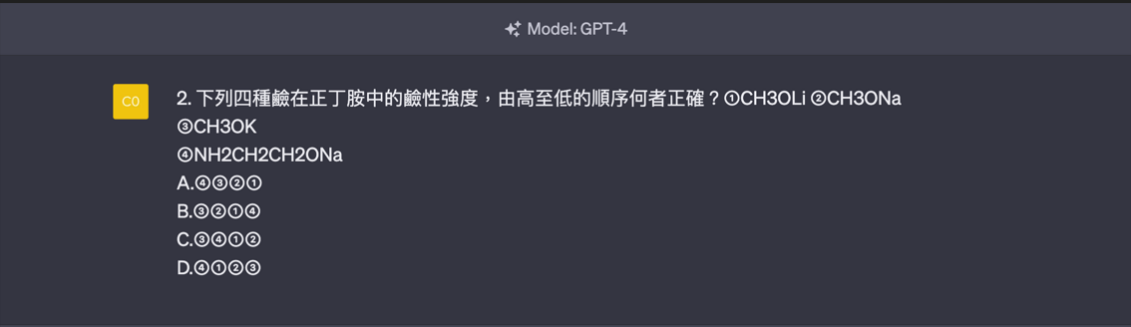
Template of a comprehension questions (multiple-choice or matching types, requiring high- level thinking).

**Figure 5.**
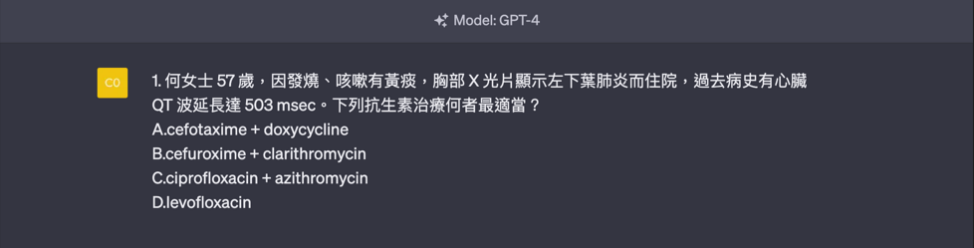
Template of a case scenario question.

The study was divided into 3 parts; the first part compared the accuracy of GPT-4 and GPT-3.5, as well as in different subjects. The second part compared the accuracy of GPT-4 and GPT-3.5 across different question types. These questions were categorized into 4 types: memory-based questions (1 correct word answer out of 4 options, low-level thinking; Figure 1), judgment questions (1 correct statement out of 4, medium-level thinking; Figure 2), reverse questions (1 incorrect statement out of 4, medium to high-level thinking; Figure 3), and comprehension questions (multiple-choice or matching types, high-level thinking; Figure 4). One pharmacist classified the questions according to these established categories and the second pharmacist reviewed the classifications. In the event of disagreement, a third pharmacist was consulted for the final decision. All pharmacists had over 10 years of experience in medical center hospitals or community teaching hospitals. The third part compared the accuracy of GPT-4 and GPT-3.5 for calculation-based and case scenario questions (Figure 5). Model testing for this study was conducted from May 10 to July 20, 2023.

### Statistical Analysis

Microsoft Excel 2019 was used to compare the accuracy rates of the 2 models. *χ*^2^ tests were used to compare the overall accuracy rates of answers obtained using GPT-3.5 and GPT-4. McNemar tests were used to compare the consistency in answers between GPT-3.5 and GPT-4, and for the calculation-based and situational question types using R software (version 4.2.2; R Foundation for Statistical Computing).

### Ethical Considerations

This study involved comparing the performance of ChatGPT-4 and ChatGPT-3.5 in the pharmacist licensing examination. It did not involve human participants. As per the guidelines of the 'Human Research Cases Exempted from Ethics Review Board' issued by the Ministry of Health and Welfare, Taiwan, this study was exempted from Ethics Review Board analysis.

## Results

### Accuracy in Different Subjects

**Figure 6.**
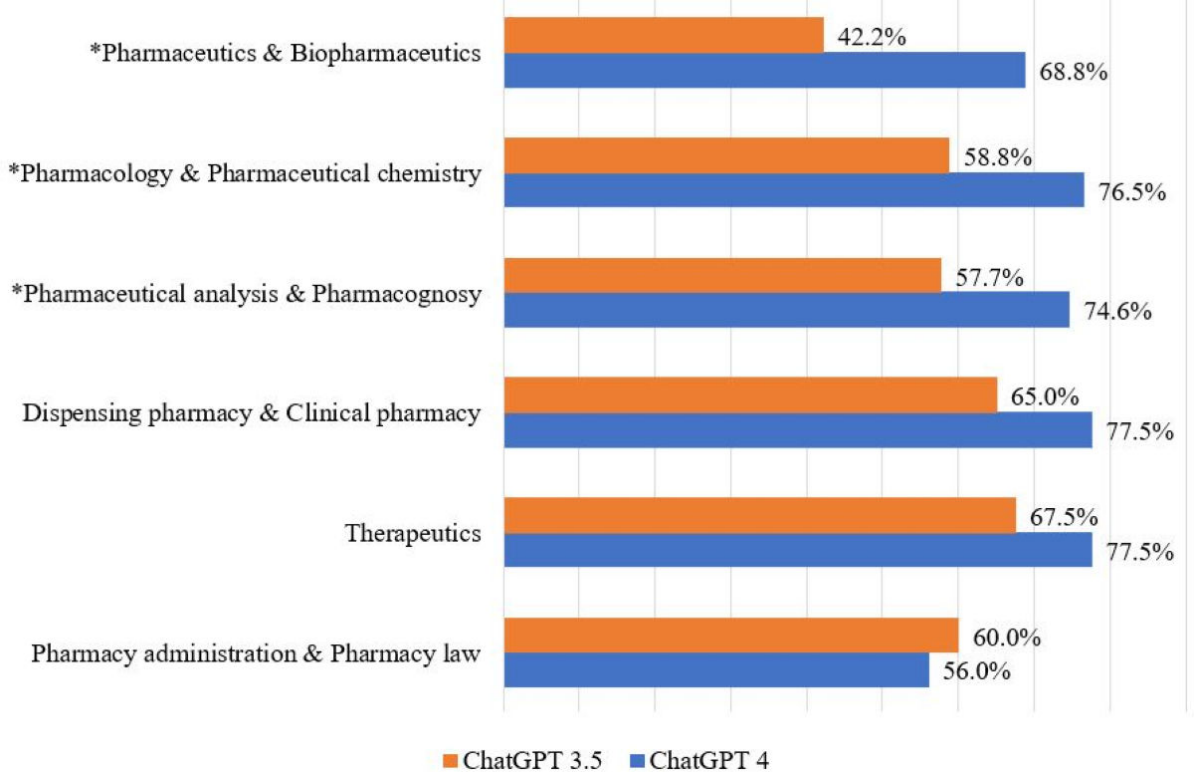
Accuracy comparison of ChatGPT-3.5 and ChatGPT-4 across different subjects. *P<.05.

In total, 203 and 210 questions were included for analysis from the first- and second-stage examinations, respectively, after excluding 37 questions containing graphical elements (N=413) (Figure 6). GPT-4 had an overall accuracy of 72.9% (301/413), easily passing the test (60% threshold) and outperforming GPT-3.5 which achieved an accuracy of 59.1% (244/413; *P*<.001). In terms of accuracy by stage, GPT-4’s overall accuracy was significantly higher than that of GPT-3.5 (73.4% vs 53.2% or 149/203 vs 108/203; *P*<.001) in basic subjects of the first stage. GPT-4 also significantly outperformed GPT-3.5 in each of the 3 basic subjects. In the clinical subjects of the second stage, GPT-4’s accuracy was higher but not statistically significant than that of GPT-3.5 (72.4% vs 64.8% or 152/210 vs 136/210; *P*=.096). In pharmacy administration and pharmacy law, GPT-4’s accuracy was lower than that of GPT-3.5 (56% vs 60% or 28/50 vs 30/50; *P*=.96). Among individual subjects, significant differences were observed in pharmacology and pharmaceutical chemistry (*P*=.02), pharmaceutical analysis and pharmacognosy (*P*=.02), and pharmaceutics and biopharmaceutics (*P*=.002). No significant differences were noted in dispensing pharmacy and clinical pharmacy (*P*=.07), pharmacotherapeutics (*P*=.10), and pharmacy administration and pharmacy law (*P*=.48).

The overall consistency among answers significantly differed between the 2 models (68%, *P*<.001), with GPT-4 showing consistent correct answers in 49.4% (n=204) of cases and consistent incorrect answers in 18.6% (n=77) of cases (Table 1).

**Table 1.** Performance comparison of consistency between ChatGPT-3.5 and ChatGPT-4.

ChatGPT-3.5 responses	GPT-4	
	Correct answers, n (%)	Incorrect answers, n (%)
Correct answer	204 (49.4)	38 (9.2)
Incorrect answer	94 (22.8)	77 (18.6)

### Accuracy in Different Question Types

Among the 413 examination questions analyzed, memory-based questions were the most common (n=254, 61.5%), followed by judgment questions (n=82, 19.9%), reverse questions (n=46, 11.1%), and comprehension questions (n=31, 7.5%). GPT-4 and GPT-3.5 did not differ significantly in terms of accuracy of answers between question types (*P*=.461 vs *P*=.18; Table 2). GPT-4 is significantly better than GPT-3.5 in memory-based questions (*P*<.001) and comprehension-based questions(*P*=.03).

**Table 2.** Accuracy comparison of ChatGPT-3.5 and ChatGPT-4 by question type.

Question type	GPT-3.5 Correct answers, n (%)	GPT-4 Correct answers, n (%)	Total, n (%)	*P* value
Memory-based questions	155 (61)	188 (74)	254 (61.5)	<.001^a^
Judgment questions	21 (45.7)	30 (65.2)	46 (11.1)	.06
Reverse questions	51 (62.6)	56 (68.3)	82 (19.9)	.41
Comprehension questions	16 (51.6)	24 (77.4)	31 (7.5)	.03^a^

a *P*<.05.

**Figure 7.**
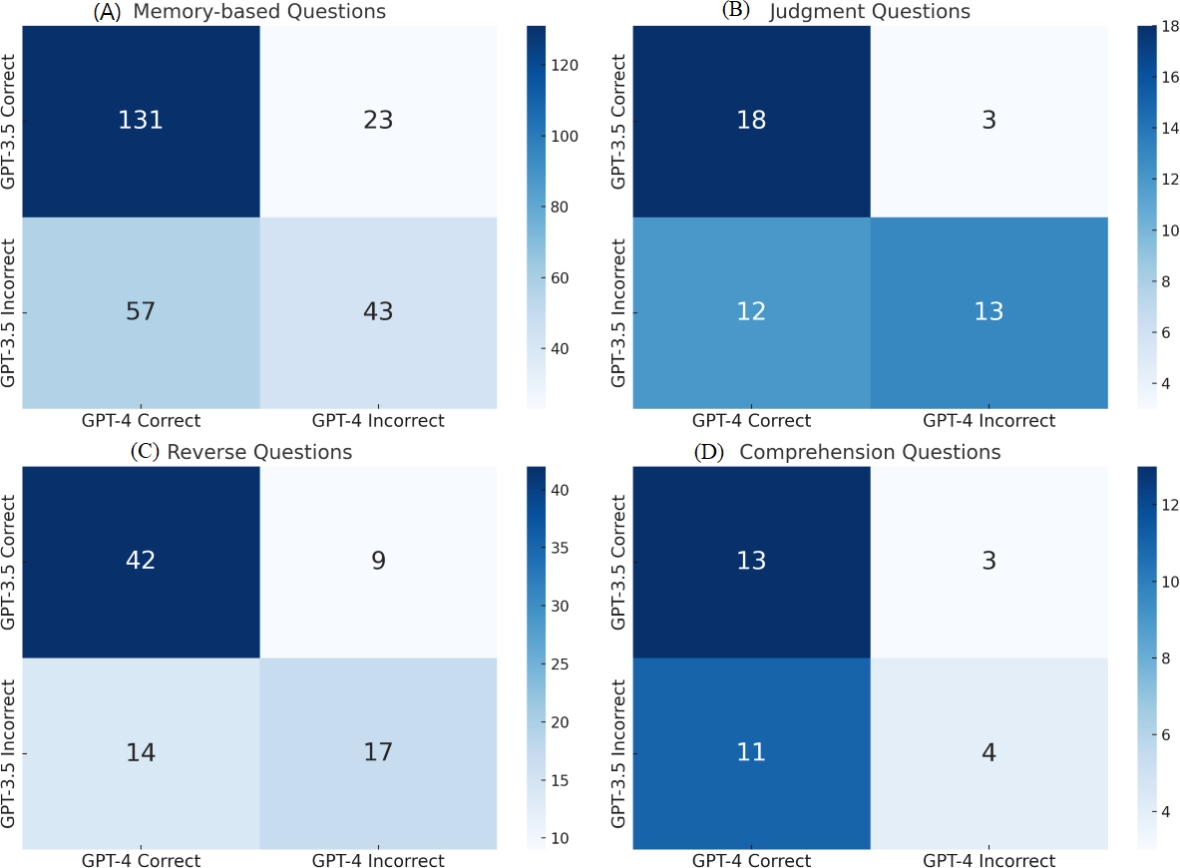
Performance comparison of GPT-3.5 and GPT-4 across question types (A) memory-based, (B) judgement, (C) reverse , and (D) comprehension. The heatmaps display the number of answers, with darker shades indicating higher counts of correct responses and highlighting model performance.

Figure 7 shows the performance comparison of GPT-3.5 and GPT-4 across question types. The data provided insights into the relative strengths and weaknesses of each model.

Further analysis of the discrepancies between the models revealed no significant difference in questions answered incorrectly by GPT-3.5 but correctly by GPT-4 (n=94) and vice versa (n=38) across the 4 question types (*P*=.27 vs *P*=.95).

For calculation-based questions, GPT-4 showed higher accuracy than that of GPT-3.5 (80% vs 40%, *P*=.03), with the most pronounced difference in pharmaceutics and biopharmaceutics subjects. In scenario-based questions, GPT-4 also outperformed GPT-3.5 in terms of accuracy (63% vs 44.4%, *P*=.41), though the difference was nonsignificant.

## Discussion

### Principal Findings

This study demonstrates that GPT-4 significantly outperformed GPT-3.5 in the Taiwan NPLE, surpassing the passing threshold, especially in basic pharmacy subjects. These subjects, which have only a 13.82% passing rate among human students, are particularly challenging. GPT-4 excelled in areas such as pharmacology, pharmaceutical chemistry, pharmaceutical analysis, and pharmaceutics, consistently providing correct answers and comprehensive explanations. Although GPT-4 also performed better than GPT-3.5 in clinical subjects such as dispensing pharmacy and therapeutics, the performance gap was narrower in these areas.

In specific subjects like pharmacodynamics, pharmacokinetics, and drug-related topics in the autonomic nervous system, GPT-4 consistently provided accurate responses, where GPT-3.5 often faltered. Additionally, GPT-4 exhibited superior accuracy in bioavailability, dosing, and pharmacokinetic calculations. However, GPT-4’s accuracy dropped in topics like herbal medicines and pharmacy law, emphasizing the need for further model refinement in these areas [30].

### Comparison with Literature

Previous studies have established that GPT-4 consistently outperforms GPT-3.5 in various medical exams, including the Australian Medical Licensing Examination [31], Canadian Radiology Examination [15], Turkish Medical Examination [32], and Japanese Medical Licensing Examination [33]. In many of these examinations, GPT-4 consistently achieved scores above 70% [34-36]. This study aligns with those findings, showing GPT-4’s superior performance in the Taiwan NPLE. Unlike prior research that focused on real-world clinical applications [37-43], this study comprehensively assessed the models across various pharmacy domains.

A study by Choi [44] reported that GPT-3.5 performed well on memory-based questions but struggled with problem-solving, whereas GPT-4 demonstrated better performance in comprehension and judgment tasks. Similarly, a radiology study suggested that GPT-4 outperformed GPT-3.5 on higher-order thinking questions but not on lower-order questions [15]. These findings slightly differ from the results of our study, where GPT-3.5 exhibited higher accuracy in both memory-based (low-level thinking) and reverse (mid-level thinking) questions. However, GPT-4 surpassed GPT-3.5 across all question types, particularly in comprehension (high-level thinking) and memory-based (low-level thinking) questions. In judgment, reverse, and comprehension questions—tasks that demand more advanced reasoning—GPT-4 demonstrated superior accuracy with fewer errors compared to GPT-3.5. Additionally, GPT-4’s ability to correct errors made by GPT-3.5 reinforces its potential as a more reliable model for pharmacy-related assessments.

**Figure 8.**
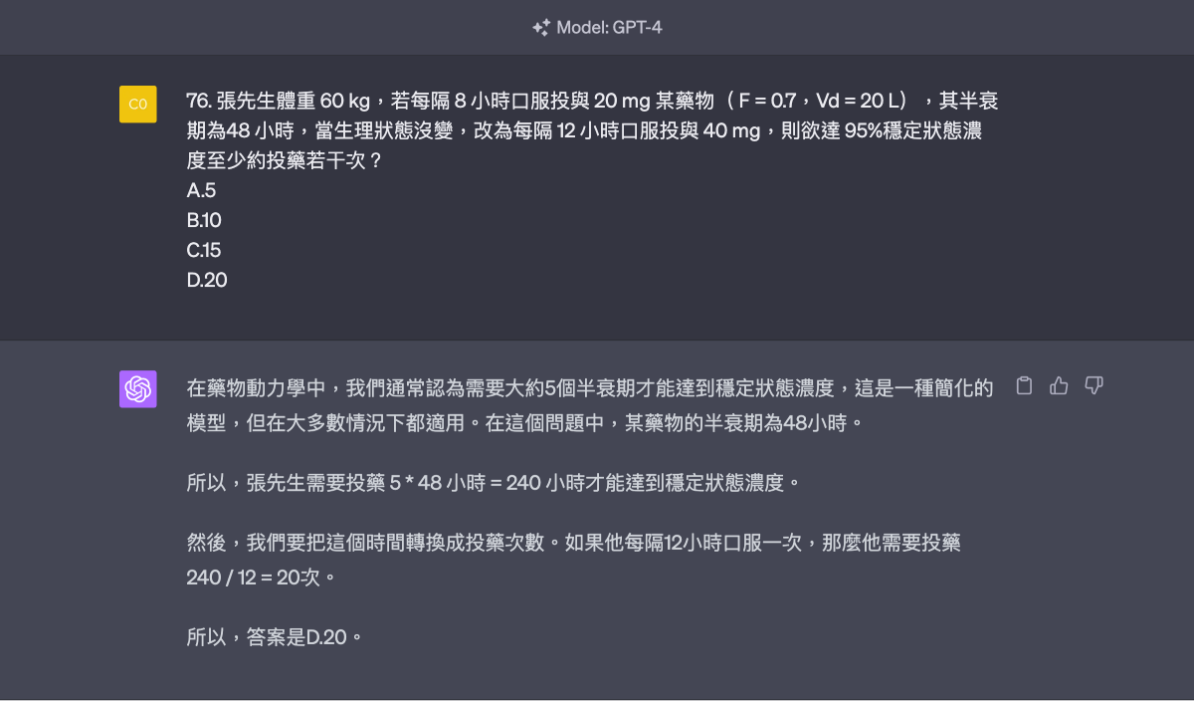
Template of the questions that GPT-4 exhibited stronger logical reasoning.

Further, GPT-4 significantly outperformed GPT-3.5 in calculation questions. While GPT-3.5 provided step-by-step explanations but often guessed the final answer—a phenomenon known as hallucination’ due to insufficient training—GPT-4 exhibited stronger logical reasoning (Figure 8) with over 80% accuracy. However, it still made errors in 20% of cases, indicating the need for needed during its use [21,45]. In clinical applications, modifying prompts has been shown to improve GPT’s accuracy [46]. For integrated analysis questions, GPT-4’s performance was slightly better than GPT-3.5, consistent with findings from a nursing licensure examination in Japan [14].

### Implications for Education

The study highlights GPT-4’s potential as an educational tool, particularly in pharmacy education. GPT-4 can offer extensive practice opportunities for pharmacy students across both basic and clinical subjects, providing both correct answers and detailed explanations [18,47] to enhance understanding. Given the lower passing rates among pharmacy students in basic subjects among that were challenging, GPT-4 could assist in individualized learning. Its strength in comprehension and integrated analysis questions makes it a valuable resource for fostering critical thinking skills.

Despite its advancements over GPT-3.5, GPT-4’s occasional inconsistencies suggest that model stability is not yet perfect. Questions correctly answered by GPT-3.5 were not always consistently answered by GPT-4. Nevertheless, GPT-4’s accuracy, approaching 80% suggests that it can serve as an effective learning supplement, provided educators guide students in minimizing potential errors. For instance, specifying clearer prompts, such as “Please do not add your own opinions”, may help mitigate hallucinations and enhance its use in educational settings.

In addition, educators should consider adjusting the format of examinations by replacing memory-based questions with comprehension questions, which can reduce the chances of guessing and better assess students’ true intelligence.

### Limitations

The primary limitation of this study is the time frame during which the models were tested (ie, from May 10 to July 20, 2023), which may affect the reproducibility of the results if retested in the future. Additionally, both GPT-3.5 and GPT-4 struggled with recognizing structural diagrams, limiting their performance in areas such as pharmaceutical chemistry and pharmacognosy. These limitations, consistent with previous research, highlight the need for cautious application of GPT models in fields that require visual recognition [11,48,49]. Additionally, the models showed poorer performance in subjects with less available training data and specific medical knowledge such as pharmacy law and traditional medicine, indicating potential biases in the models’ training. We suggest that future efforts in model development should focus on incorporating more diverse and comprehensive data to reduce such biases.

### Conclusions

This study demonstrates that GPT-4 outperforms GPT-3.5 in the Taiwan NPLE, particularly in pharmacy expertise, calculation ability, and situational case studies, with a notable advantage in basic subjects. It is recommended that GPT-4 be applied in clinical pharmacy practice (ie, patient education, drug consultation) and pharmacy education, particularly to support self-directed learning. However, given its limitations, caution is advised when integrating GPT-4 into clinical settings and educational programs. Future research should focus on refining prompts, improving model stability, integrating medical databases, and enhancing comprehensive questions to evaluate student competence more effectively while minimizing the chance of guessing correct answers.

## Abbreviations

GPT-3.5: ChatGPT-3.5
GPT-4: ChatGPT-4
NPLE: National Pharmacist Licensing Examination
USMLE: United States Medical Licensing Examination
